# Nurturing gut health: role of m6A RNA methylation in upholding the intestinal barrier

**DOI:** 10.1038/s41420-024-02043-x

**Published:** 2024-06-03

**Authors:** Shuaijie Wang, Yuzhong Yang, Xiaohan Jiang, Xiang Zheng, Qiufang Wei, Wenbin Dai, Xuemei Zhang

**Affiliations:** 1 Guilin Medical University, Guilin, Guangxi China; 2 Department of Pathology, Affiliated Hospital of Guilin Medical University, Guilin, Guangxi China; 3 Department of Pathology, Liuzhou People’s Hospital Affiliated to Guangxi Medical University, Liuzhou, Guangxi China

**Keywords:** Epigenetics, Immunology, Microbiology

## Abstract

The intestinal lumen acts as a critical interface connecting the external environment with the body’s internal state. It’s essential to prevent the passage of harmful antigens and bacteria while facilitating nutrient and water absorption. The intestinal barriers encompass microbial, mechanical, immunological, and chemical elements, working together to maintain intestinal balance. Numerous studies have associated m6A modification with intestinal homeostasis. This review comprehensively outlines potential mechanisms through which m6A modification could initiate, exacerbate, or sustain barrier damage from an intestinal perspective. The pivotal role of m6A modification in preserving intestinal equilibrium provides new insights, guiding the exploration of m6A modification as a target for optimizing preventive and therapeutic strategies for intestinal homeostasis.

## Facts

- The intestinal barriers serve the crucial roles of pathogen isolation, disease resistance, immune response regulation, nutrient absorption facilitation, and mutual influence on each other’s stability.
- m6A modification affects intestinal microbiota and immune microenvironment.
- m6A modification plays a pivotal role in preserving the stability of the intestinal barrier and modulating the intestinal immune system.

## Open questions

- How do the four components of the intestinal barrier interact and regulate each other?
- How does m6A modification work in intestinal barrier homeostasis?
- Is there a mutual influence between m6A modification and intestinal barrier homeostasis?
- How can m6A modification exert new therapeutic effects by regulating intestinal homeostasis?

## Introduction

The gastrointestinal tract, as the largest and most critical interface where organisms interact with the external environment, serves as a primary defense against external microorganisms and diseases [1, 2]. The intestinal barriers, comprising microbial, mechanical, chemical, and immune components, function together to uphold the stability of the intestines. They serve the crucial roles of pathogen isolation, disease resistance, immune response regulation, nutrient absorption facilitation, and mutual influence on each other’s stability [1, 3].

Recent research has revealed varying degrees of intestinal barrier dysfunction in patients with autoimmune diseases, colorectal cancer (CRC), and microbial infections. This dysfunction manifests as changes in gut microbiota diversity and abundance, increased intestinal mucosal permeability, and compromised immune responses [4–7]. Notably, fluctuations in N6-methyladenosine (m6A) modification levels have also been observed in these conditions [8–10]. m6A modification, a prevalent internal RNA chemical modification involving the methylation of adenosine at the N6 site, has emerged as a key player in regulating intestinal homeostasis [11]. The impact of m6A modification extends to various physiological and cellular processes, including circadian rhythm regulation, stem cell self-renewal and differentiation, responses to heat shock and DNA damage, spermatogenesis, T-cell homeostasis, inflammation, and cancer initiation [12–20].

**Fig. 1:**
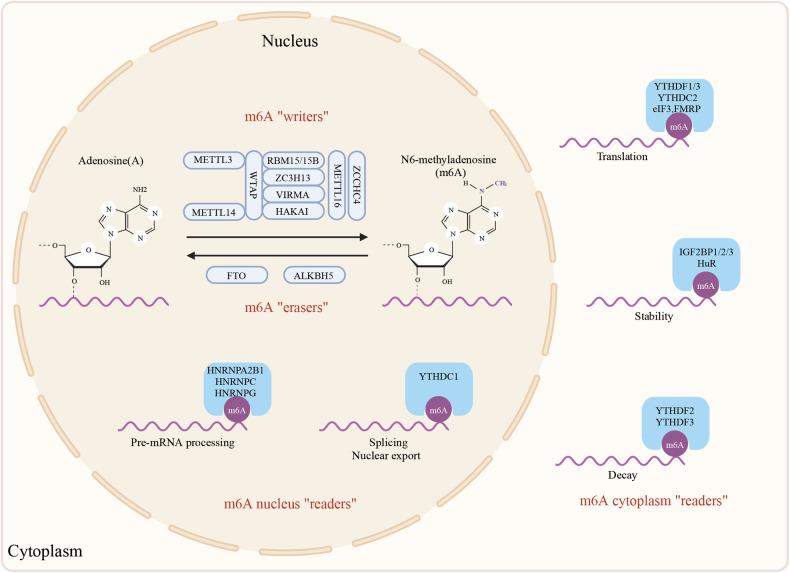
The molecular mechanisms of m6A modification. m6A modification is a dynamic and reversible chemical modification. The methyltransferase complex, primarily composed of METTL3, METTL14, and WTAP, acts as the “Writers” responsible for catalyzing the formation of m6A. Demethylases, including FTO and ALKBH5, function as “Erasers” by catalyzing m6A demethylation. RNA-binding proteins, such as YTHDF1-3, YTHDC1-2, and IGF2BPs, serve as “Readers” that recognize and bind to m6A-modified mRNA transcripts. They play crucial roles in regulating RNA processing, stability, translation, and splicing in an m6A-dependent manner (Created with BioRender.com).

The m6A modification primarily occurs near stop codons and 3’-untranslated regions (3’-UTRs). It is dynamically regulated by methyltransferases and demethylases, operating on the RRACH consensus sequence (R = G or A; H = A, C, or U), thereby influencing gene expression at post-transcriptional levels [21–23]. m6A modification significantly impacts various aspects of RNA metabolism in mammals, including RNA stability, splicing, translation, nucleation, and RNA-protein interactions [24–27]. The regulation of m6A modification involves three classes of proteins working in concert (Fig. 1). The m6A writers has the capacity of catalyzing RNA methylation. In eukaryotes, the METTL3/METTL14 complex installs most m6A marks on mRNA [28]. METTL3, the catalytic subunit, transfers a methyl group from S-adenosylmethionine (SAM) to the N6 position of adenosine residues in RNA. Concurrently, METTL14 acts as an allosteric activator, augmenting METTL3’s catalytic activity. Investigations indicate that the METTL3/METTL14 complex is aided by proteins like WTAP, RBM15, VIRMA, ZC3H13, and HAKAI, recruiting the complex to its target locations [29–33]. Another methyltransferase, METTL16, with a C-terminal vertebrate conserved region (VCR) and an N-terminal methyltransferase domain (MTD), primarily targets specific structural motifs in RNA, such as U6 snRNA, for m6A modification [34]. ZCCHC4, a recently discovered rRNA methyltransferase, catalyzes m6A modification at position 4220 on 28 S rRNA, enhancing ribosome assembly and translation efficiency. This modification influences crucial cellular processes such as cell proliferation and tumor progression [35]. The m6A erasers has the capacity of removing m6A from RNA. “Erasers” encompass the demethylases fat mass and obesity-associated protein (FTO) and alkb homologue 5 (ALKBH5), responsible for removing m6A modifications from RNA [36, 37]. “Readers” are m6A-specific binding proteins that recognize and bind m6A sites, influencing various biological processes related to RNA, such as degradation, processing, splicing, and translation [38]. These m6A-binding proteins can be classified into nuclear and cytoplasmic “readers” based on their mechanisms of action. Nuclear “readers”, such as YTH domain-containing protein 1 (YTHDC1), heterogeneous nuclear ribonucleoproteins (HNRNPs; including HNRNPC, HNRNPA2B1, HNRNPG), play roles in mRNA alternative splicing, gene silencing, nuclear mRNA export, and RNA structure modification [22, 30, 39, 40]. Cytoplasmic “readers”, such as YTHDF1-3, insulin-like growth factor 2 mRNA-binding proteins (IGF2BPs; including IGF2BP1-3), fragile X mental retardation protein (FMRP), and eukaryotic initiation factor 3 (eIF3), influence mRNA stability, translation, and degradation [41–43]. Uniquely, human antigen R (HuR) is identified as a reader enlisted by METTL3 [44]. Furthermore, HuR constricts METTL14 expression by immediately binding to its promoter [45, 46].

Numerous RNA m6A methylation regulators participate in diverse physiological processes, although many remain unidentified. In this paper, we explore the pivotal roles of m6A modification in influencing intestinal barrier function. Furthermore, we evaluate the benefits and drawbacks of studying m6A modification in the context of intestinal barriers, its potential applications in assisting with diagnosis and treatment, and prospects for future research.

## Microbial barrier and m6A

The intestinal microbial barrier consists of a diverse community of microorganisms in the gut, including bacteria, viruses, archaea, and fungi. This microbiota outnumbers human cells and plays a crucial role in nutrient absorption and maintaining the intestinal barrier function [47]. They achieve this by producing metabolites like short-chain fatty acids, secondary bile acids, and tryptophan metabolites [48–52]. The microbiota also stimulates epithelial cells to express immune recognition receptors like toll-like receptors (TLRs) and NOD-like receptors, promoting immune defenses such as antimicrobial peptide (AMP) secretion and mucus production to prevent foreign microorganism invasion [53]. The adhesion of bacteria to the intestinal mucosa helps maintain intestinal homeostasis by initiating immune and chemical defenses while regulating intracellular reactive oxygen species levels [54–56]. An imbalance in the gut microbiota is closely linked to various diseases, including metabolic diseases, infectious diseases, CRC, inflammatory bowel disease (IBD), celiac disease, and irritable bowel syndrome [57–64]. Fecal microbiota transplantation has shown favorable results in alleviating active ulcerative colitis (UC) in patients and has provided strong evidence for improving disease activity and colonic mucosal inflammation [65–67]. The human intestinal tract hosts trillions of crucial microorganisms that contribute to nutrition, infection risk reduction, and autoimmune disorder prevention [68, 69]. Research has highlighted the significant influence of both gut microbiota and m6A modification on the development of cancer, hinting at the possible interplay between these two factors within the disease’s progression [70]. Notably, the presence of m6A methylation on bacterial genomes has been identified, which suggests a potential link between m6A modification and the integrity of the gut microbiota barrier [71–73].

### Gut microbiota regulates host m6A modification

**Fig. 2:**
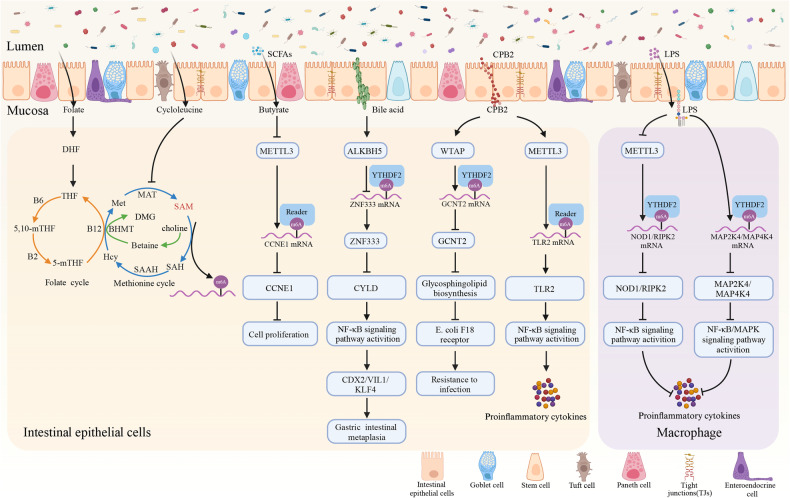
The gut microbiota regulates m6A modification. The gut microbiota plays a role in m6A modification levels by influencing SAM through the production of folate and cycloleucine, which participate in the folate and methionine cycles. SCFAs, secondary bile acids, and CPB2 mediate the m6A modification of CCNE1, ZNF333, GCNT2, and TLR2, leading to the regulation of intestinal epithelial cell proliferation, metaplasia, anti-infection mechanisms, and their ability to modulate inflammatory responses. LPS produced by the microbiota regulates the homeostasis of the intestinal barrier through an m6A-dependent manner, affecting macrophage production of inflammatory factors (Created with BioRender.com).

Numerous investigations have demonstrated the significant impact of the microbiome on host m6A mRNA modification (Fig. 2). Recent research has focused on comparing germ-free (GF) mice and specific pathogen-free (SPF) mice in terms of total m6A/A ratios, transcriptomic m6A structures, and localization in various organs. These investigations have unveiled variations in m6A levels within the colon, liver, and brain of SPF and GF mice, which were associated with the expression of m6A writer and eraser enzymes. Notably, in the brain, where the microbiome is absent, there is a considerable reduction in m6A modification. Interestingly, m6A patterns in the brains of GF mice more closely resemble those in embryonic mouse brains than those in age-matched SPF mice, indicating rapid m6A modification adaptation to microbiota interactions shortly after birth [74]. Another study demonstrated that the gut microbiota influences the m6A transcriptome in the cecum and liver of mice. Researchers compared m6A modifications in cecal transcripts between SPF mice and GF mice, identifying 440 distinct m6A peaks across 312 transcripts. When GF mice were colonized with SPF mouse intestinal contents, they exhibited similar patterns of the most abundant gut bacterial genera observed in SPF mice. Additionally, administering antibiotics to SPF mice to deplete their gut microbiota resulted in m6A peaks in SPF mice that closely resembled those found in GF mice. Researchers also identified two commensal bacteria, *Akkermansia muciniphila,* and *Lactobacillus plantarum*, that influence host m6A modifications in the cecum and liver. Both of these bacteria were found to impact cellular growth, proliferation, and even cell death [75]. Collectively, these outcomes establish that the gut microbiota as an entire or unique commensal control host epi-transcriptomic m6A RNA methylation levels.

A clear consensus exists that m6A modification is introduced by a methyltransferase complex, utilizing SAM as a methyl donor and removed by demethylases using ferrous iron and α-ketoglutarate as cofactors to oxidize the N-methyl chain of m6A [76, 77]. SAM and α-ketoglutarate levels govern these processes in eukaryotic cells. Interestingly, the gut microbiota regulates SAM and α-ketoglutarate availability by orchestrating the folate cycle, methionine cycle, and tricarboxylic acid (TCA) cycle. This interplay affects m6A modification dynamics. The gut microbiota not only produces SAM but also influences the gut microbiome composition, subsequently impacting SAM supply [78, 79]. This interplay affects mRNA m6A levels, contributing to mRNA stability and the expression of tight junction protein ZO-1, crucial for cellular barrier integrity [80].

Notably, a series of metabolites synthesized by the gut microbiota, including compounds like betaine, folate, and cycloleucine, have been identified as chemical agents capable of functioning both as methyl donors and methylation inhibitors. These metabolites assume a critical role in the modulation of m6A modifications across diverse contexts, spanning from porcine adipocytes to murine models and zebrafish [81–83]. A recent investigation suggests that butyrate, a short-chain fatty acid produced by bacteria, enters the TCA cycle to augment levels of α-ketoglutarate. This enhancement, in turn, inhibits the m6A modification level in 3′-UTR (1511-1515nt) of CCNE1 mRNA mediated by METTL3, further reducing the expression of cyclin E1 (CCNE1) and successfully inhibiting the development of CRC [84]. Furthermore, the presence of vitamin B12 was found to be indispensable for the synthesis of SAM. Deficiency in vitamin B12 led to a global reduction in m6A levels, subsequently contributing to abnormal neurological functions in mice [85]. In addition to these findings, bile acids have been shown to upregulate the m6A modulator ALKBH5 in gastric epithelial cells, subsequently elevating zinc finger protein 333 (ZNF333) levels through an m6A-YTHDF2-dependent mechanism. This intricate process has been implicated in the development of gastric intestinal metaplasia [86]. Collectively, these investigations underscore the pivotal role of gut microbiota-derived metabolites in the regulation of m6A levels.

Commonly, lipopolysaccharide (LPS), an endotoxin produced by Gram-negative bacteria, can be released into the intestine due to bacterial infection and escalation of inflammatory reactions [87]. It may affect the host’s m6A modification under oxidative stress. An Investigation uncovered that LPS-stimulated macrophages exhibited reduced total m6A levels and METTL3 expression. Knocking down METTL3 facilitated the mRNA expression and stability of NOD1 and RIPK2. This interference by METTL3 resulted in the prevention of NOD1 and RIPK2 mRNA degradation, which is managed by YTHDF2. Consequently, this modulation led to the activation of the NOD1 pathway and subsequently heightened the inflammatory response induced by LPS in macrophages [88]. Another research shows that YTHDF2 can mitigate LPS-induced inflammation in mouse macrophages. LPS stimulation increases YTHDF2 expression. Knocking down YTHDF2 elevates MAP2K4 and MAP4K4 mRNA levels by stabilizing transcripts, subsequently activating the MAPK and NF-κB pathways. This triggers the release of proinflammatory cytokines, intensifying the inflammatory response [89]. Remarkably, not only does the endotoxin produced by Gram-negative bacteria affect the degree of intestinal m6A modification, but the exotoxin of Gram-positive bacteria can also regulate intestinal m6A levels. The *Clostridium perfringens* beta2 (CPB2) toxin is a key pathogenic toxin produced by *Clostridium perfringens*, responsible for causing intestinal disorders such as diarrhea in animals and humans [90–92]. Researchers observed a notable surge in overall m6A RNA methylation levels in porcine intestinal epithelial (IPEC-J2) cells after exposure to CPB2 toxin. This m6A modification was associated with CPB2-triggered inflammatory and antiviral responses, potentially influencing them via gene expression modulation within the Wnt signaling pathway [90, 91]. Moreover, CPB2 treatment elevated m6A and METTL3 levels in IPEC-J2 cells. Mechanistically, METTL3 overexpression activated the TLR2/NF-κB pathway, exacerbating CPB2-induced inflammatory responses in these cells [92].

The mechanism through which the gut microbiota controls the host’s m6A methylome at the molecular level is becoming increasingly clear. Recently, research has indicated that microbiota originating from either IBD patients or mice can decrease the expression of YTHDC1 in macrophages situated in the intestines, consequently expediting the development of IBD in animal models [93]. Building upon these discoveries, it can be deduced that the gut microbiota orchestrates host m6A modifications through a multifaceted approach: furnishing m6A modification donors and generating various microbial metabolites or toxins.

### Host m6A affects gut microbiota

The gut microbiome is shaped by various factors, including host genetics, environmental elements like diet, habits, immune status, infections, and medications [94]. Mounting data suggests that host m6A modification impacted gut microbiota by regulating inflammatory responses within the gastrointestinal tract [11, 95]. Intriguingly, conditional knockdown of METTL14 in T cells has been found to trigger spontaneous colitis in mice [11, 95]. METTL14 deficiency in T cells not only induces colitis but also leads to a decrease in *Bacteroidales* families *S24-7* and *Lachnospiraceae*, alongside an increase in *Bacteroidaceae*, *Helicobacteraceae*, *Deferribacteraceae*, and *Enterobacteraceae* when compared to control mice [95]. Inversely, FTO-deficient mice exhibit a distinct bacterial profile that suppresses inflammation, featuring higher levels of *Lactobacillus*, especially *Lactobacillus murinus* and *Lactobacillus reuteri*, and lower levels of *Porphyromonadaceae* and *Helicobacter* compared to controls [96]. Additionally, YTHDF1 enhances *A. muciniphila* colonization, augmenting mice’s anti-inflammatory properties by increasing the expression of FOXP3 and elevating 5-HT levels [97]. These findings highlight the potential of altering host m6A modification to influence gut microbiota.

## Mechanical barrier of the intestine and m6A

The intestinal mechanical barrier relies on three key components: mucous secretion, epithelial cells, and tight junctions [2]. Mucous secretion, predominantly by epithelial and goblet cells, forms a protective layer composed of water, proteins, lipids, and electrolytes [98]. This layer prevents direct contact between pathogens and the epithelium, with mucins and immune components playing pivotal roles in maintaining barrier integrity [98, 99]. Nestled beneath the mucus layer, the gut epithelium is home to a varied spectrum of cell types, encompassing enterocytes, goblet cells, enteroendocrine cells, Paneth cells, and M cells. These cells, functioning as the primary defense, not only physically obstruct the intrusion of harmful substances into the body but also facilitate the absorption of nutrients [100–102]. In addition, these cells possess regenerative capabilities and contribute to the secretion of mucus, AMPs, and secretory immunoglobulin A [103]. These substances form the fundamental basis of the gut’s physical, chemical, and immune barriers.

**Fig. 3:**
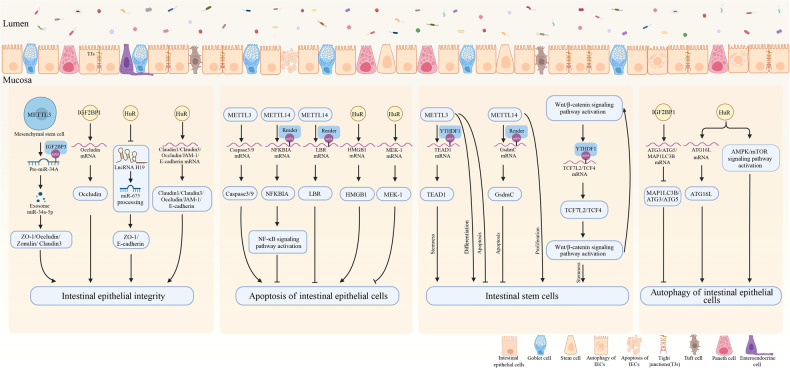
The effect of m6A modification on the intestinal mechanical barrier. m6A modification regulates intestinal epithelial integrity by affecting tight junction protein expression, and it influences intestinal epithelial cell survival by impacting apoptosis, autophagy, stem cell proliferation, as well as differentiation, eventually maintaining intestinal mechanical barrier homeostasis (Created with BioRender.com).

Tight junctions (TJs) are particularly crucial. These protein complexes create continuous loops in the apical and lateral regions of epithelial cells, reinforcing the mechanical barrier [4, 104]. They consist of integrin proteins such as Claudin, Occludin, ZO-1, and Cingulin, regulating ion and nutrient transport and preventing the translocation of antigens, pathogens, and toxins [104, 105]. Disruption of tight junctions is linked to various intestinal diseases [106]. The orchestrated collaboration of mucous secretion, epithelial cells, and tight junctions upholds the mechanical barrier’s selective permeability for nutrients and resistance against pathogens [4, 107]. This barrier is susceptible to environmental factors, medications, diet, microbiota, immune conditions, and infections that may compromise its integrity. Consequently, a compromised barrier can lead to immune cell recruitment, cytokine release, and disrupted homeostasis. The emerging role of m6A modification in modulating these mechanical barriers, especially in the context of inflammatory disorders, signifies its importance in maintaining intestinal health (Fig. 3).

### Intestinal epithelial cell survival and m6A

Intestinal stem cells (ISCs), which dwell at the bottom of crypts, are essential for intestinal homeostasis and regeneration by preserving the completeness and viability of epithelial tissues through a precisely regulated balance of proliferation and differentiation [108]. The overall influence of m6A mRNA methylation on stem cell biology is complex; however, it is emerging as a critical modulator in regulating stem cell pluripotency. Knocking down METTL3 or METTL14 weakens ISCs stemness, triggering apoptosis, disrupting stem cell development, and undermining intestinal barrier homeostasis [11, 109, 110]. Interestingly, METTL3 deficiency doesn’t affect ISCs proliferation differently from METTL14 deficiency [110]. Investigators found that deleting METTL14 from gut epithelial cells leads to significant growth retardation and premature mortality in mice. Furthermore, the depletion of METTL14 in IECs disrupts colonic epithelial morphogenesis, resulting in the development of spontaneous colitis and systemic inflammation. Subsequently, they observed that depleting METTL14 notably reduces vital mucin-producing goblet cells. Moreover, METTL14 deficiency decreases MUC2 and ITF/TFF3, compounding tight junction dysfunction and harming the epithelial barrier. Fundamentally, METTL14-mediated m6A modification of GsdmC mRNA plays a vital role in the viability of colonic Lgr^5+^ ISCs, thereby ensuring the accurate development of colonic mucosal architecture in mice [109]. YTHDF1 was deemed nonessential for both intestinal development and regeneration. It assumes a pivotal role in driving the advancement of intestinal diseases by actively influencing ISCs properties [111, 112]. Research has shown that YTHDF1 is highly expressed in ISCs and is upregulated through Wnt signaling at the translational level. While largely nonessential for intestinal homeostasis under physiological conditions, it plays a vital role in regulating ISCs properties during intestinal epithelial regeneration. YTHDF1 intestinal conditional knockout mice subjected to systemic γ-irradiation exhibit impaired recovery, with a significant reduction in both regenerating crypt quantity and size. YTHDF1 deletion also suppresses Wnt-driven tumorigenesis. This is linked to a decrease in the translation of Wnt signaling effectors, including TCF7L2/TCF4, resulting from the deletion of YTHDF1. This leads to the inactivation of Wnt signaling and subsequent decline in stemness [111]. Additionally, YTHDF1 contributes to the maintenance of ISCs by upregulating the expression of transcriptional-enhanced associate domain 1 (TEAD1) through an m6A-dependent mechanism [113].

A recurring pattern of dysregulated IEC death, compromise of the intestinal barrier, and ensuing inflammation forms the central mechanism in chronic intestinal inflammatory diseases. Research has found that METTL14 plays a critical role in maintaining colonic epithelial cell homeostasis by restricting cell death. Deficiency of METTL14 has been shown to promote cell apoptosis and inhibit the formation and function of mature colonic epithelial cells, leading to the development of dextran sulfate sodium (DSS)-induced colitis. Mechanistically, METTL14 sustains colonic epithelial cell survival by enhancing the stability of Nfkbia mRNA and limiting TNF-induced cell death through NF-κB activity [11]. In a Drosophila model, the lack of METTL14 shortened lifespan and compromised intestinal integrity. Further cellular experiments revealed that reducing METTL14 led to a decrease in m6A modification in lamin B receptor (LBR) mRNA. This instability in LBR mRNA triggered intestinal cellular senescence [114]. In LPS-stimulated IECs, suppressing the expression of METTL3 resulted in inhibited cell apoptosis, accompanied by a significant reduction in cleaved-caspase3 and cleaved-caspase9 protein levels [115]. HuR also plays a critical role in the regulation of IEC apoptosis. On the one hand, HuR competitively binds to high mobility group box 1 (HMGB1) with miR-29a-3P, thereby facilitating apoptosis of colonic epithelial cells in the 2,4,6-trinitro-benzene sulfonic acid (TNBS)-induced model of UC [116]. On the other hand, HuR enhances the stability of mitogen-activated protein kinase kinase 1 (MEK-1) mRNA and promotes its translation, subsequently protecting IECs against TNF-α/cycloheximide-triggered apoptosis [117].

Autophagy is a conserved degradation mechanism that plays a crucial role in maintaining normal cellular and organismal metabolism [118]. Intestinal tissue from IBD patients had higher levels of IGF2BP1 expression than normal tissues. Research has revealed that eliminating IGF2BP1 from IECs diminishes the repercussions of DSS-induced colonic damage. This outcome can be attributed to IGF2BP1’s role as a negative regulator of autophagy, achieved through its interaction with transcripts of pivotal autophagy genes such as MAP1LC3B, ATG5, and ATG3, resulting in the inhibition of autophagy in IECs [119]. HuR, whose expression is lower in IBD patients, also aids in the survival of IECs through the regulation of autophagy. However, its regulatory role in autophagy is entirely opposite to that of IGF2BP1. Deficiency of HuR inhibits autophagy and exacerbates intestinal inflammation in LPS-stimulated IECs. The mechanism by which HuR promotes autophagy is associated with the activation of AMPK/mTOR pathway [120]. In addition, the knockout of HuR from IECs reduces ATG16L1 levels, disrupting autophagy and thereby compromising the defensive functions of IECs [121].

These findings suggest that m6A plays a crucial role in maintaining the survival and differentiation of ISCs, as well as regulating processes such as apoptosis and autophagy. These mechanisms indirectly influence the repair of the intestinal mucosal barrier, highlighting the significance of m6A in intestinal homeostasis.

### Intestinal tight junctions and m6A

Extensive research has unequivocally highlighted the pivotal role of m6A regulatory factors in upholding the integrity of the intestinal mechanical barrier. Regulatory elements within the m6A framework can either bolster or hinder the stability of molecules associated with intestinal tight junctions, directly impacting the efficiency of the intestinal mechanical barrier. This mechanism plays a critical role in preserving intestinal homeostasis.

In previous studies, it has been reported that METTL3/IGF2BP3-mediated m6A methylation of pre-miR-34a increases the stability of pre-miR-34a, leading to the upregulation of miR-34a-5P in mesenchymal stem cells. This upregulation of miR-34a-5P plays a role in enhancing intestinal barrier function and alleviating oxygen-glucose deprivation/reoxygenation-induced intestinal damage. This effect is achieved through the upregulation of tight junction proteins, such as ZO-1, Occludin, Zonulin, and Claudin-3 [122]. IGF2BP1 also participates in the regulation of the intestinal mechanical barrier in UC. Loss of IGF2BP1 exacerbates DSS-induced colitis in mice, suggesting a protective role for this protein. Mechanistically, IGF2BP1 maintains intestinal barrier function by stabilizing Occludin mRNA and up-regulating Occludin expression [123]. However, whether these RNA-binding proteins regulate the intestinal mucosal mechanical barrier through an m6A-dependent mechanism remains to be further investigated. HuR, a crucial m6A recognition protein, is found to have decreased levels in the colonic epithelium of IBD patients compared to controls [124, 125]. HuR competes with CIRP for binding to the 3’-UTR of Claudin1 mRNA, resulting in the upregulation of Claudin1 expression. This upregulation enhances the integrity of intestinal cell barriers, thus mitigating damage and reducing cell apoptosis [125]. Furthermore, HuR can protect against intestinal barrier dysfunction by inhibiting lncRNA H19’s facilitative role in miR-675 processing, thereby promoting the expression of ZO-1 and E-cadherin [126]. Numerous studies have also demonstrated that HuR directly interacts with the 3’-UTR of tight junction proteins, such as Occludin, JAM-1, Claudin-1, Claudin-3, and E-cadherin mRNAs. This interaction promotes translation and stability, thereby strengthening the mechanical integrity of the intestinal barrier [127–130].

## Immune barrier of the intestine and m6A

**Fig. 4:**
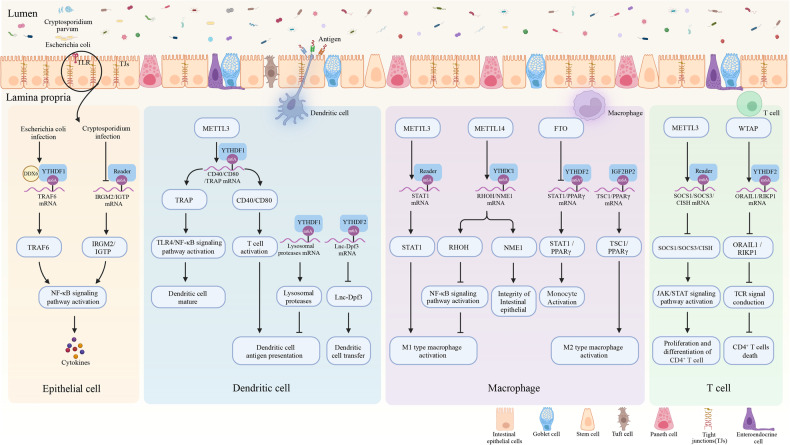
The effect of m6A modification on the intestinal immune barrier. Microbial and parasitic infections mediate the production of inflammatory factors and damage to intestinal epithelial cells through the m6A modification pathway. The m6A modification is essential for dendritic cell maturation, antigen presentation, and cell transfer processes. It also plays a significant role in macrophage polarization and innate immune responses. m6A modification also influences T cell proliferation, differentiation, and apoptosis to regulate adaptive immune responses (Created with BioRender.com).

The intestine is the most massive immunological organ in the human body. What looks particularly impressive is that the intestinal immune system must monitor vast mucosal surfaces and changing landscapes, protecting us from pathogenic insults while limiting inflammatory responses against the resident commensal microbiota and providing tolerance to food antigens, which establish and maintain intestinal homeostasis. Immune barrier dysfunction results in prolonged immunological activation, which is a core feature of innate and adaptive immune disorders such as autoimmune illnesses, microbial infection, and cancer. Accumulating evidence reveals that m6A modification is essential for a successful immune response [131, 132] (Fig. 4).

### The Role of m6A modification in innate immune system

Innate immunity is a component of the immune system that is present in all multicellular organisms, providing a non-specific first line of defense against pathogens [133, 134]. It is powerful, but it does not confer long-lasting immunity. The innate immune system consists of receptors that detect microorganisms’ conserved structural motifs, as well as a range of cell types involving neutrophils, dendritic cells (DCs), monocytes, macrophages, and natural killer cells, which permit quick and efficient inflammatory responses against microbial invasion [134]. In addition, although epithelial cells are not innate immune cells, they participate in innate immune defense [135]. These defenses not only serve as the primary line of defense against infection, but also assist bridge to specific immunological protection via T-cell induction and stimulation and the adaptive immune system [133].

The earliest step in innate immunity involves pathogen recognition, facilitated by a range of pattern-recognition receptors, particularly plasma membrane receptors like TLRs, and cytosolic sensors, including RIG-I-like receptors and NOD-like receptors in IECs [135]. m6A modification influences host innate immune antigen recognition. A study indicated that m6A methylation statuses play a pivotal role in shaping the innate defense of intestinal epithelium against cryptosporidial infection. Mechanistically, cryptosporidial infection reduces the m6A modification of immune-related genes, such as IRGM2 and IGTP, leading to elevated expression levels of both genes. IRGM2 and IGTP are crucial components of the immune response, and their increased expression plays a significant role in combating the infection [136]. YTHDF1 is essential for IECs’ defense against bacterial infection by regulating Traf6 expression, a pivotal regulator of TLRs and NF-κB signaling. Specifically, YTHDF1 facilitates the transcription and expression of m6A-modified Traf6 mRNA in a DDX60-dependent manner, ultimately impairing IECs’ defense against bacterial infection [137].

DCs, vital in linking innate and adaptive immunity, shift from immune tolerance as immature DCs to immune activation as mature DCs, with dysregulation leading to undesired immune responses and disorders [138]. Emerging evidence underscores the pivotal role of m6A methylation in driving DCs activation and function, holding promise for therapeutic interventions. Recent research has revealed that METTL3-mediated mRNA m6A methylation promotes the activation and function of DCs. Specifically, when METTL3 is depleted in DCs, it impairs their maturation process, leading to reduced expression of key molecules such as CD40 and CD80. Consequently, the ability of DCs to stimulate T-cell responses is compromised both in vitro and in vivo. The m6A modification facilitated by METTL3 plays a crucial role in enhancing the translation of CD40, CD80, and TIRAP transcripts in DCs. This, in turn, promotes T-cell activation and reinforces the TLR4/NF-κB signaling-induced cytokine production in DCs [139]. Likewise, inhibiting the METTL3 gene in dendritic cells results in immaturity and extends allograft survival [140]. Furthermore, YTHDFs can also participate in immune response events by regulating DC function. In DCs, inhibiting YTHDF2 leads to the upregulation of lnc-Dpf3 expression. Subsequent investigations have unveiled that the reader protein YTHDF2 recognizes and selectively targets the m6A modifications of lnc-Dpf3 at positions 3,812–3,815nt and 3,852–3,855nt near its 3’ end, resulting in the degradation of lnc-Dpf3. Importantly, mice with deficient lnc-Dpf3 expression specifically in DCs exhibit heightened inflammation [141]. The lack of YTHDF1 expression in classical dendritic cells heightened the in vivo cross-presentation of tumor antigens and the cross-priming of CD8^+^ T cells. Mechanistically, YTHDF1 recognized transcripts encoding lysosomal proteases that carried m6A modifications, facilitating the translation of lysosomal cathepsins. The absence of YTHDF1 led to the inhibition of cathepsins, thereby enhancing cross-presentation by DCs and contributing to the anti-tumor immune response [142].

Macrophages are innate immune cells with the ability to engulf pathogens, present antigens to activate T cells, and regulate inflammation through specific molecular and cellular pathways [143]. They can polarize into two distinct types, classically activated (M1) and alternatively activated (M2) macrophages, depending on microenvironmental cues [144]. In the context of intestinal immunological disorders, the balance between M1 and M2 macrophages is disrupted, making macrophage polarization an attractive therapeutic target. Recent research has demonstrated that m6A modification plays a crucial role in regulating macrophage activation and polarization. METTL3, a key methyltransferase, promotes the polarization of M1 macrophages by directly methylating STAT1 mRNA. This modification increases mRNA stability and consequently enhances STAT1 expression [145]. Similarly, the FTO/YTHDF2 axis modulates M1 and M2 macrophage activation by regulating mRNA decay of STAT1 and PPARγ. Knockdown of FTO reduces the stability of STAT1 and PPARγ mRNA, suppressing both M1 and M2 macrophage polarization, while YTHDF2 knockout has the opposite effect [146]. Furthermore, YTHDC1 has been identified as a crucial factor in resolving inflammatory responses and restoring the integrity of the colonic epithelial barrier in the context of IBD. Recent research has revealed that METTL14 mediates the m6A modification of Rhoh mRNA at positions 603–607 nt and 616–620 nt, as well as Nme1 mRNA at positions 497-501nt and 590-594nt. Moreover, YTHDC1 promotes Rhoh expression to suppress inflammatory responses and enhances Nme1 expression to strengthen the integrity of the colonic epithelial barrier, dependent on the METTL14/m6A pathway [93]. Additionally, the evidence highlights the significant involvement of IGF2BP2 in macrophage activation during the development of colitis induced by DSS. Researchers observed the presence of IGF2BP2 in approximately half of CD68^+^ macrophages from patients with UC. Depletion of IGF2BP2 resulted in an increase in the M1-like macrophage phenotype and promoted the development of DSS-induced colitis. Furthermore, IGF2BP2 facilitates the transition from M1 to M2 activation by targeting TSC1 and PPARγ through an m6A-dependent mechanism [147]. Taken together, m6A regulates macrophage activation and polarization in a complicated and significant way.

### The role of m6A in adaptive immune system

The intestinal epithelium and underlying lamina propria serve as the main sites for the accumulation of adaptive immune cells in the intestine. Adaptive immunity primarily focuses on eradicating specific infections through the activation of antigen-specific T and B cells, ultimately leading to the development of enduring immunological memory for the targeted antigen. Numerous studies have underscored the pivotal role of m6A modification regulation in shaping adaptive immune responses [95, 148, 149].

In particular, the crucial role of m6A in governing T cell homeostasis has been unveiled. m6A modification regulates the differentiation of Naive CD4^+^ T cells by targeting the mRNA of signaling molecules in the IL-7/STAT5/SOCS pathway, thereby maintaining the dynamic balance of the immune system. The methyltransferase METTL3 plays a central role in this process. Specifically, specific knockout of Mettl3 in CD4^+^ T cells promotes the expression of IL-7 signaling inhibitory molecules, such as SOCS1, SOCS3, and CISH, through the m6A modification pathway. This inhibition suppresses the activation of the JAK/STAT signaling pathway, disrupting the differentiation of naive CD4^+^ T cells and inhibiting the development of colitis in a T cell transfer-induced colitis model [150]. Additionally, research has demonstrated that WTAP, involved in N6-adenosine methylation of mRNAs, controls T cell receptor (TCR) signaling and the survival of T cells. Mice with T cells lacking the WTAP gene were found to develop colitis at an early age. This deficiency leads to the spontaneous activation and reduced abundance of CD4^+^ and CD8^+^ T cells through a cell-intrinsic mechanism. Moreover, it interferes with TCR-induced expansion. In this context, WTAP mediates the m6A methylation of Orai1 and Ripk1 mRNAs, equipping CD4^+^ T cells with regulatory capabilities to control cell death and influence the survival of T cells following TCR stimulation. This mechanism may explain the observed dysfunctionality in WTAP-deficient T cells in vivo and provides potential insights into understanding the underlying mechanisms [151].

## Chemical barrier and m6A

The intestinal chemical barrier, complementing the mechanical barrier, is crucial for maintaining intestinal microecology balance and connecting innate immunity with the microbiota. Comprised of gastric acid, bile, digestive enzymes, lysozyme, and AMPs, it serves as a defense against foreign antigens [4, 152]. AMPs, like defensins (α-, β-, θ-types), lysozyme C, phospholipases (sPLA2), and C-type lectins (REG3), originate mainly from Paneth cells. These cationic proteins exhibit broad-spectrum antibacterial activity, disrupting bacterial membranes [153–155]. Defensins, in particular, hinder bacterial cell wall synthesis through interactions with lipid II [156]. Moreover, they activate CCR6-positive dendritic cells, neutralize exotoxins, and regulate water and salt absorption by IECs [157–159]. This conditional interplay underscores the significance of AMPs and lysozyme in the intestinal context.

However, damage and insufficiency of the chemical barrier in the intestine render it highly susceptible to various threats. Recent research on m6A modification has introduced a fresh perspective on potential treatments. There was compelling evidence that the production of β-defensin in response to Enterotoxigenic Escherichia coli K88 (E. coli K88) infection was regulated by the cellular m6A methyltransferase METTL3. Observation revealed that METTL3-deficient IECs exhibited reduced β-defensin production in an m6A-dependent manner. Furthermore, their findings suggest that following E. coli K88 infection, METTL3 enhances the transcription of GPR161 through its methyltransferase activity, thereby triggering the expression of β-defensin [160]. In another study, it was noted that mice with a targeted knockout of HuR in intestinal cells exhibited a decrease in the number of Paneth cells and a reduction in lysozyme granules. Mechanistic investigations revealed that HuR regulates Paneth cells by modulating the membrane localization of TLR2. This regulation occurs through HuR’s binding to the coding region of CNPY3, subsequently leading to an increase in CNPY3 expression at the posttranscriptional level [124].

## Potential therapeutic implications of m6A modification in intestinal disease

With the development of research technology, the delivery of several medicinal drugs using nanoparticles (NPs) has shown promise. Fascinatingly, an accurate and secure alternative to conventional treatment has been provided by NPs-based drug delivery systems with colon targeting capabilities [161]. Recent research underscores the potential of vesicle-like nanoparticle-encapsulated ALKBH5-siRNA in suppressing CRC growth by bolstering the antitumor immune response [162]. The utilization of vesicle-like nanoparticles-siRNA to target YTHDF1 not only enhances the effectiveness of anti-PD1 therapy in microsatellite instability-high CRC but also overcomes anti-PD1 resistance in microsatellite stable CRC [163]. Some investigators harnessed m6A modification to develop a dual-stimulation T1 imaging-based nanoparticle system (DAS) for precise cancer therapy. Within DAS, superparamagnetic quenchers (Fe_3_O_4_ NPs) and paramagnetic enhancers (Gd-DOTA complexes) are connected via a molecular device activated by m6A-caged, Zn^2+^-dependent DNAzymes. Once tumor cells uptake DAS, the overexpression of FTO triggers m6A demethylation, consequently activating DNAzymes to cleave the substrate strand and release Gd-DOTA labeled oligonucleotides. This process facilitates precise laser irradiation to induce apoptosis in tumor cells [164]. These advances highlight the exciting prospects of nanotechnology and m6A modification in revolutionizing colon-related disease treatments.

Compound Adenosine (1), identified through high-throughput docking into the SAM binding site and protein X-ray crystallography, marks a significant stride in applying m6A modification to clinical settings. It represents the first structurally characterized small molecule inhibitor of METTL3, operating as a SAM-competitive inhibitor with an IC50 value of approximately 500 µM. This discovery holds promise for the clinical translation of m6A modification [165]. Subsequently, METTL3 inhibitors have been continuously developed by designing compounds that act as competitors of the cosubstrate SAM [166]. Utilizing allosteric inhibitors like CDIBA, researchers have achieved a reversible and noncompetitive interaction with the METTL3-METTL14 complex, ultimately leading to the reduction in METTL3 enzymatic activity [167]. Notably, natural product, such as quercetin, has recently been identified as METTL3 inhibitors [168]. While the application of targeting m6A modificantion in intestinal tumors and inflammation-related diseases have not gained widespread recognition in research, recent developments in research targeting METTL3 in other contexts have ignited new hope. For instance, Cpd-564, a METTL3 inhibitor, has demonstrated superior protective effects against cisplatin- and ischemia/reperfusion-induced renal injury and inflammation compared to S-adenosyl-l-homocysteine [169]. STM2457, another highly selective METTL3 inhibitor, has been confirmed to significantly reduce the growth of acute myeloid leukemia while promoting both differentiation and apoptosis [170]. The latest studies revealed that METTL3 activates the m6A-BHLHE41-CXCL1/CXCR2 pathway, recruiting myeloid-derived suppressor cells within the immune microenvironment of CRC. Targeting METTL3 through METTL3–sgRNA or the chemical inhibitor STM2457 enhances the efficacy of anti-PD-1 treatment [171]. Furthermore, Sun et al. uncovered that METTL3 triggers m6A methylation of DCP2, instigating the degradation of DCP2. Consequently, this process promotes mitochondrial autophagy through the Pink1-Parkin pathway, contributing to chemotherapy resistance in small-cell lung cancer (SCLC). Notably, the METTL3 inhibitor STM2457 exhibits the capacity to reverse chemoresistance in SCLC [172]. These findings suggest the significant potential for targeting m6A modification in the development of clinical anti-tumor drugs. Additionally, meclofenamic acid (MA) was identified as a highly selective inhibitor of FTO. It is a non-steroidal, anti-inflammatory drug that competes with FTO binding for the m6A-containing nucleic acid [173]. Another potential FTO inhibitor is entacapone, which has shown the ability to reduce body weight and lower fasting blood glucose concentrations in diet-induced obese mice [174].

## Discussions and perspectives

m6A RNA modification plays a pivotal role in preserving the stability of the intestinal barrier and modulating the intestinal immune system. With the rapid advancement of next-generation sequencing technologies, an abundance of data has unveiled extensive m6A modifications within bacterial genomes and various microbiota, each displaying distinct methylation patterns and levels. Remarkably, research has demonstrated the capacity of the gut microbiota to influence host m6A modification levels, providing compelling evidence for microbial regulation of host m6A modifications. On one hand, the composition of the gut microbiota can impact the modification of host m6A, functioning either as a whole or as an isolated symbiote to regulate host m6A modification levels. Moreover, the gut microbiota coordinates host m6A modification by supplying m6A-modified donors and generating an array of microbial compounds and toxins. On the other hand, the mechanical components of m6A may induce changes in the gut microbiota by controlling pH, bile acid metabolism, and inflammatory responses, thereby impacting gut microenvironmental homeostasis. The reciprocal connection between the host and gut bacteria, influencing gene expression and microbiome composition, underscores the presence of signaling molecules acting as mediators [175, 176]. However, the intricate molecular mechanisms through which host m6A modification manipulates the gut microbiota necessitate further investigation. Current studies have suggested that m6A RNA modification may impact the metabolism of non-coding RNAs, such as miRNA, circRNA, and lncRNA, while also participating in chromatin remodeling and histone modification [177]. In light of these findings, m6A may serve as a conductor in the orchestration of host-microbiome interactions, working in synergy with non-coding RNAs, chromatin remodeling, and histone modifications [178].

In the intestine, billions of bacteria coexist within the colon without breaching the intestinal mucosa. Alongside microbial homeostasis, a robust mucosal barrier, comprising mechanical and chemical components, safeguards the intestinal surface. The mechanical barrier is composed of a tightly sealed layer of epithelial cells and mucus on the surface of the epithelium. m6A modification exerts its influence on the stability of mRNAs encoding tight junction-associated genes, while also directly or indirectly impacting epithelial cell apoptosis and regeneration, thereby modulating the integrity of the intestinal epithelial barrier. Mucin is the functional main body of the mechanical barrier mucus layer, mainly composed of secreted mucin and transmembrane mucin, both of which play crucial functions in the integrity of the intestinal mucosal barrier and pathogen invasion. The m6A modification represents an innovative approach for further regulating mechanical barriers by modulating mucin levels. MUC2 is the primary secreted mucin found on the surface of epithelial cells. MUC2’s rich O-glycans serve as attachment sites for bacterial adhesins. Not only that, it also functions as a key energy source for symbiotic bacteria [179]. MUC1 is the primary transmembrane mucin located on the apical surface of epithelial cells. The protruding structure on the surface can prevent mucosal infections from attaching to underlying cell receptors. Its cytoplasmic tail domain has the potential to trigger a series of signal transduction processes [180–182]. Without mucus, bacteria can directly contact the epithelium, translocate, and activate the submucosal immune system, eliciting a strong immunological response. Furthermore, m6A modification may impact the secretion of defensins, although its effects on mucin and immunoglobulin A remain to be elucidated.

Intestinal epithelial cells manage the gastrointestinal mucosa’s natural defense against microbial illnesses. m6A modification can modulate immune-related gene expression by changing microbial antigen recognition in the intestinal epithelium, consequently influencing the fine regulation of epithelial anti-infection. Dendritic cells, specialized antigen-presenting cells in the immune system, have the ability to perceive their surroundings and initiate adaptive immune responses. The m6A modification regulates the maturation, migration, antigen presentation, and immune response of dendritic cells. However, the specific role and mechanism of m6A modification remain unclear. For instance, it is uncertain how different pathways tightly regulate the extent and duration of inflammatory reactions, how to achieve a balance between immune activation and tolerance, and how to govern the development of various CD4^+^ T helper cell subsets. In mammalian intestines, macrophages inside tissues are critical for intestinal homeostasis and function [183]. Intestinal macrophages are highly adaptable. On the one hand, they produce an overabundance of inflammatory mediators, which prolongs the inflammatory response. On the other hand, macrophages not only form a defense line against infections, but also stimulates tissue repair and regeneration following injury [93, 184]. m6A modification can impact the polarization of M1 and M2 macrophages, influencing the inflammatory response and intestinal epithelial integrity. Further research is necessary to determine the impact of m6A modification on the migration, mobility, and balance of M1 and M2 macrophages. Metabolites produced by the microbial community, in particular, are thought to have immune regulatory capabilities, which govern macrophage plasticity [184]. T cell homeostasis is essential for the maintenance of T cell reserves and serves as the foundation for adaptive immunity. The m6A modification impacts intestinal adaptive immunity by influencing T cell proliferation, differentiation, and survival, but the exact mechanism is uncertain. There is evidence that it is related to the influence of m6A modification on RNA dynamics during T-cell development [18].

The four integral components of the intestinal barrier interact and mutually regulate to maintain intestinal homeostasis. Establishing a sustainable mutualism between the host and the intestinal barrier is crucial for preserving intestinal homeostasis. While the relationship between m6A modification and the intestinal barrier has been investigated across various human disorders, comprehensive conclusions are still pending. Further research is essential to unravel the precise role of m6A-mediated interactions among the intestinal barrier components (gut microbiota, physical defense, immune defense, chemical defense) in the pathogenesis of diverse pathological conditions. These findings have the potential to unveil novel therapeutic strategies for addressing a broad spectrum of diseases, including autoimmune disorders, microbial infections, celiac disease, and CRC.
